# Factors Driving Readmissions in Tibia and Femur Fractures

**DOI:** 10.1155/2015/974543

**Published:** 2015-03-10

**Authors:** Alexander Chern, Sarah E. Greenberg, Rachel V. Thakore, Vasanth Sathiyakumar, William T. Obremskey, Manish K. Sethi

**Affiliations:** The Vanderbilt Orthopaedic Institute Center for Health Policy, Vanderbilt University, 1215 21st Avenue S., MCE, South Tower, Suite 4200, Nashville, TN 37232, USA

## Abstract

As the American healthcare system shifts towards bundled payments, readmissions will become a measure of healthcare quality. The purpose of this study was to characterize readmission trends and factors influencing readmission in patients with diaphyseal femur and tibia fractures. Through a retrospective chart review, all patients who presented to a level 1 trauma center from 2004 to 2006 were evaluated. By using current procedural terminology codes, 1,040 patients with diaphyseal tibia or femur fractures fixed by IMN were identified. 645 patients were included for analysis. 30-day, 60-day, and 90-day readmission rates were compared with fracture type, reason for readmission, and basic demographic information. The 60-day readmission rate for open tibia fractures (14.8%) was significantly higher than the 60-day readmission rate for closed tibia fractures (8.0%) (*p* = 0.037). When comparing reasons for 60-day readmissions, 50% of closed fractures were readmitted due to infection, while the other 50% needed additional surgery. 91.7% of open fractures readmitted in 60 days were due to infection. In a bundled payment system, orthopedic trauma must gain insight into drivers of readmission to identify those at risk for readmission and design effective healthcare plans for these patients.

## 1. Introduction

A significant portion of healthcare costs stem from hospital readmissions. With nearly one in five Medicare patients returning to the hospital within a month of discharge, the United States Government considers patient readmissions a serious problem in an expensive and inefficient healthcare system. The Medicare Payment Advisory Commission (MedPAC) estimates that 12% of Medicare patients are readmitted for potentially avoidable reasons [1]. Nearly 2 million Medicare patients are readmitted within 30 days of release annually, costing Medicare $17.5 billion in additional hospital bills. Since hospital readmissions reflect hospital care quality and account for billions of dollars in annual Medicare costs, reducing readmissions among Medicare beneficiaries has become a key priority for physicians [1].

To combat these unnecessary rehospitalizations, CMS has started to levy a maximum penalty of 1% of Medicare payments to hospitals for excessive readmissions for patients with heart failure, heart attack, and pneumonia [1]. In fiscal year 2015, this list will be expanded to at least four additional conditions, including chronic obstructive pulmonary disease, coronary artery bypass graft surgery, percutaneous transluminal coronary angioplasty, and other conditions the government deems appropriate [1]. With CMS's institution of episode-based payment, hospitals must also assume the costs for all care provided up to 30 days after discharge, including all readmissions occurring in that time [2]. Moreover, operative management of fractures contributes significantly to surgical expenses, with musculoskeletal conditions accounting for approximately $510 billion in treatment costs per year [3–5]. One study by Nacke et al. found that 30-day readmission rates are as high as 80.4% for orthopedic spine fractures and 58.3% for total joint arthroplasty, while another study by Hahnel et al. demonstrated a 3-month readmission rate of 19.0% for operative hip fractures [6, 7].

As the US healthcare system shifts toward new policies that no longer reimburse hospitals for perioperative readmission and even penalize hospitals for unnecessary readmission, it is important for orthopedic trauma surgeons to develop tools to assess the risk of postoperative readmission for both optimizing patient safety and minimizing costs. Readmissions following orthopedic procedures are an important factor in evaluating the cost-effectiveness of these procedures, and establishing a system to reduce risk of readmission has already been shown to be successful in reducing costs for the healthcare system [8, 9]. With an increasing emphasis on reducing healthcare costs by predicting readmission rates and creating an appropriate healthcare plan for the patient, it is critical to gain insight into the readmission trends of different fractures.

In this study, we investigate the postoperative readmission trends for tibia and femur fractures and explore the factors influencing readmission.

## 2. Methods 

After receiving IRB approval, all patients who presented to a level I trauma center from Jan. 1, 2004, to Dec. 31, 2006, with a diaphyseal tibia or femur fracture fixed by IMN were identified using CPT codes and the institution's orthopaedic database. A total of 1,040 patients were identified. Patient charts were reviewed to identify and select isolated cases where there was only a single fracture that required operative fixation with no other organ injury (*n* = 645), while patients with multiple injuries were excluded from the study. The charts of the selected 645 patients with isolated fractures were reviewed for readmission information and basic demographic information, including age, gender, initial length of stay, and fracture type (open versus closed). Readmission information included reason for readmission and whether or not the patient was readmitted within 0 to 30 days, 0 to 60 days, or 0 to 90 days after initial discharge from the hospital. Only rehospitalizations related to the original surgery were included for this study. Patients were grouped into fracture type (tibia or femur) and were further categorized by whether the fracture was open or closed. Readmitted patients were also grouped into the following readmission categories: postoperative infection (either for incision and drainage or antibiotics), postoperative surgical revision (for removal of hardware, nonunion, or revision), and nonoperative medical conditions (UTIs, PNAs, hypotension, anemia, etc.). A multivariate analysis controlling for age, gender, race, and ASA score was performed to determine if there is a statistically significant difference in 30-, 60-, and 90-day readmission rates for open and closed fractures within each type of fracture (tibia or femur). To calculate *p*-values, *t*-tests were used due to large sample sizes.

## 3. Results

There were 645 selected patients, 489 with femur fractures (367 closed versus 122 open) and 156 with tibia fractures (75 closed versus 81 open). Demographic information for these groups is shown in Table 1.

**Figure 1:**
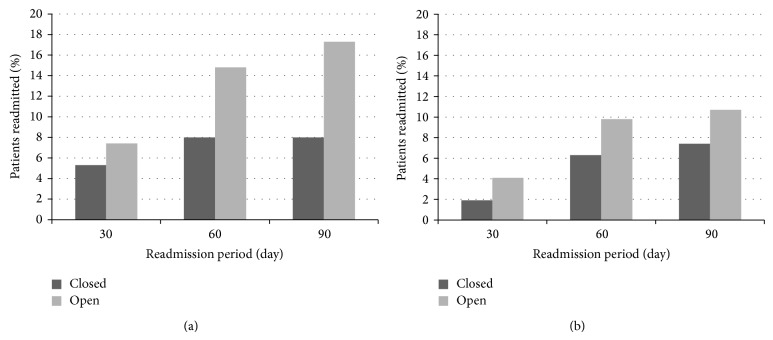
(a) 30-Day, 60-day, and 90-day readmission rates for isolated diaphyseal tibia fractures. (b) 30-Day, 60-day, and 90-day readmission rates for isolated diaphyseal femur fractures.* Note*. All orthopaedic trauma surgery patients who presented with a diaphyseal tibia or femur fracture to VUMC between January 1, 2004, and December 31, 2006, were included in this analysis. (a) compares 30-day, 60-day, and 90-day readmission for closed tibia and open tibia fractures. (b) compares 30-day, 60-day, and 90-day readmission for closed femur and open femur fractures.

**Figure 2:**
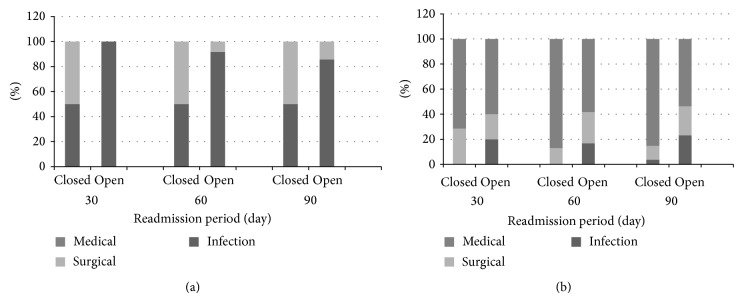
(a) Patients readmitted for isolated diaphyseal open and closed tibia fractures classified by reason for readmission. (b) Patients readmitted for isolated diaphyseal open and closed femur fractures classified by reason for readmission.* Note*. All orthopaedic trauma surgery patients who presented with a diaphyseal tibia or femur fracture to VUMC between January 1, 2004, and December 31, 2006, were included in this analysis. Reasons for readmissions were classified as “infection” (postoperative infection), “surgical” (postoperative surgical revision), and “medical” (nonoperative medical conditions such as UTIs, PNAs, hypotension, and anemia).

Of the patients with tibia fractures (*n* = 156), 48.1% had closed fractures (*n* = 75), while 51.9% had open fractures (*n* = 81). The average length of stay for the index hospitalization was 3 days for both open (SD ± 2.5) and closed (SD ± 1.5) fractures. There was an overall 12.8% (*n* = 20) readmission rate within the overall 90-day perioperative period. Open tibia fractures demonstrated a 90-day readmission rate of 17.3% (*n* = 14), while closed tibia fractures had a 90-day readmission rate of 8.0% (*n* = 6). These rates were not significantly different after controlling for several factors (age, gender, race, and ASA score) (*p* = 0.084). Of those readmitted within 90 days in the closed tibia fracture group (*n* = 6), 50.0% (*n* = 3) were due to postoperative infection, 50.0% (*n* = 3) were due to surgical revision, and 0.0% (*n* = 0) were due to nonoperative medical conditions. Of those readmitted within 90 days in the open tibia fracture group (*n* = 14), 85.7% (*n* = 12) were due to postoperative infection, 14.3% (*n* = 2) were due to surgical revision, and 0.0% (*n* = 0) were due to nonoperative medical conditions. When breaking readmission down further into separate 30- and 60-day readmission periods, closed fractures had a 30-day readmission rate of 5.3% (*n* = 4) compared to 7.4% (*n* = 6) for open fractures, but these rates were not significantly different (*p* = 0.6). Of those readmitted within 30 days in the closed tibia fracture group (*n* = 4), 50.0% (*n* = 2) were due to postoperative infection, 50.0% (*n* = 2) were due to surgical revision, and 0.0% (*n* = 10) were due to nonoperative medical conditions. Of those readmitted within 30 days in the open tibia fracture group (*n* = 6), 100% (*n* = 6) were due to postoperative infection. However, the 60-day readmission rate for open fractures (14.8%, *n* = 12) was significantly higher than the 60-day readmission rate for closed fractures (8.0%, *n* = 6) (*p* = 0.037). When comparing reasons for 60-day readmissions, 50% (*n* = 3) of closed fractures were readmitted due to infection, while the other 50% (*n* = 3) required additional surgery for removal of hardware, nonunion, or revision. 91.7% (*n* = 11) of the open fractures readmitted in 60 days were readmitted for infection issues and 8.3% (*n* = 1) were readmitted due to surgery. These results are shown in Table 2(a) and represented graphically in Figures 1(a) and 2(a).

Of the patients with femur fractures (*n* = 489), 75.1% had closed fractures (*n* = 367), while 24.9% had open fractures (*n* = 122). The average length of stay for the index hospitalization was about 5 days for both open (SD ± 5.8) and closed (SD ± 5.25) fractures. There was an overall 8.2% (*n* = 40) readmission rate within the overall 90-day perioperative period. Open femur fractures demonstrated a 90-day readmission rate of 7.4% (*n* = 27), while closed femur fractures had a 90-day readmission rate of 10.7% (*n* = 13). There was no significant difference between these rates (*p* = 0.25). Of those readmitted within 90 days in the closed femur fracture group (*n* = 27), 3.7% (*n* = 1) were due to postoperative infection, 11.1% (*n* = 3) were due to surgical revision, and 85.2% (*n* = 23) were due to nonoperative medical conditions. Of those readmitted within 90 days in the open femur fracture group (*n* = 13), 23.1% (*n* = 3) were due to postoperative infection, 23.1% (*n* = 3) were due to surgical revision, and 53.8% (*n* = 7) were due to nonoperative medical conditions. When breaking readmission down further into separate 30- and 60-day readmission periods, closed fractures had a 30-day readmission rate of 1.9% (*n* = 7) compared to 4.1% (*n* = 5) for open fractures, but these rates were not significantly different (*p* = 0.18). Of those readmitted within 30 days in the closed femur fracture group (*n* = 7), 0.0% (*n* = 0) were due to postoperative infection, 28.6% (*n* = 2) were due to surgical revision, and 71.4% (*n* = 5) were due to nonoperative medical conditions. Of those readmitted within 30 days in the open femur fracture group (*n* = 5), 20.0% (*n* = 1) were due to postoperative infection, 20.0% (*n* = 1) were due to surgical revision, and 60.0% (*n* = 3) were due to nonoperative medical conditions. Closed fractures had a 60-day readmission rate of 6.3% (*n* = 23), while open fractures had a 60-day readmission rate of 9.8% (*n* = 12). However, there was no significant difference between these rates (*p* = 1.9). Of those readmitted within 60 days in the closed femur fracture group (*n* = 23), 0.0% (*n* = 0) were due to postoperative infection, 13.0% (*n* = 3) were due to surgical revision, and 87.0% (*n* = 20) were due to nonoperative medical conditions. Of those readmitted within 60 days in the open femur fracture group (*n* = 12), 16.7% (*n* = 2) were due to postoperative infection, 25.0% (*n* = 3) were due to surgical revision, and 58.3% (*n* = 7) were due to nonoperative medical conditions. These results are shown in Table 2(b) and graphically in Figures 1(b) and 2(b).

## 4. Discussion

Open tibia fractures have significantly higher rates of 60-day readmission than closed fractures. For 60-day tibia fracture readmissions, infection drove the vast majority of readmissions for open injuries, while closed injury readmissions were equally driven by infection and need for surgery. However, the study was unable to establish a significant difference in 30-day and 90-day readmission rates for closed and open tibia fractures. In addition, the study was unable to establish a significant difference in 30-day, 60-day, and 90-day readmission rates for closed and open femur fractures. These results suggest that open fractures do not drive readmission for femur fractures. The majority of femur fracture readmissions were driven by nonoperative medical conditions.

In a bundled payment system, where orthopedic surgeons may be penalized for readmissions and where readmission is a measure of quality, it is crucial that we have a better understanding of the drivers of care and readmission. Since hospital readmissions are a significant contributor to hospital costs, gaining insight into the readmission trends of different fractures can help orthopedic trauma surgeons identify patients at greatest risk for readmission, allowing for the appropriate allocation of resources to reduce hospital costs and the design of appropriate healthcare plans to maximize patient care. While previous studies have documented complication rates for diaphyseal femur and tibia fractures, no study has yet explored readmission rates for these particular fractures or the impact of open fractures on readmission. Moreover, the readmission rates for diaphyseal femur and tibia fractures can be directly correlated with the new episode-of-care concept, since the study investigated the rate of readmissions within 30-, 60-, and 90-day periods following initial discharge. CMS's institution of episode-based payment mandates that hospitals must assume the costs for all care provided up to 30 days after discharge, including readmissions [1].

Additional studies with different approaches in methodologies and categorization of patient readmission should be used to establish which types of readmission are most common and if other variables can predict a patient's risk for being readmitted for these reasons as well. For example, Hahnel et al. divide readmissions following hip fracture surgery into orthopedic causes, medical causes, surgical causes, and rehabilitation failure causes [7]. Being able to identify specific reasons for readmission will enable hospitals to further improve allocation of resources to minimize readmission and hospital costs and maximize patient care [1]. Though we accounted for demographic factors such as age, race, gender, and ASA score in our statistical analyses, there may be other unidentified covariates we can include that may influence 90-day patient readmission. Additionally, our study did not control for the socioeconomic status of each patient, which is found to be a risk factor for trauma injuries [10]. According to Zhou et al., socioeconomic factors, such as unemployment and minimal education, increase the risk of developing postoperative complications [11].

Future directions include investigating additional fracture types, especially common ones such as clavicle and hip fractures, to characterize readmission rates for the various injuries seen in orthopedic trauma. Factors besides the open or closed nature of a fracture should be evaluated for their effect on postoperative readmission. Different classification systems, such as the Gustilo Open Fracture Classification System (see Table 1) and the Müller AO Classification of Fractures, can be useful because injury localization and severity influence the orthopedic trauma surgeon's choice of treatment and the patient's outcome, which will affect readmission rates [12].

Our study is the first of its kind to investigate readmission rates for diaphyseal femur and tibia fractures, as well as the impact of open fractures on readmission. We demonstrate that open tibia fractures have significantly higher rates of 60-day readmission than closed fractures. For 60-day tibia fracture readmissions, infection drove the vast majority of readmissions for open injuries, while closed injury readmissions were equally driven by infection and need for surgery. We also demonstrated that there is no significant difference in 30-day, 60-day, and 90-day readmission rates for femur fractures, thus suggesting that open fractures do not drive readmission in femur fractures. Instead, the majority of femur fracture readmissions were driven by nonoperative medical conditions. As changes to the US healthcare system result in eliminating reimbursements for postoperative readmission, it is crucial for physicians to gain insight into readmission trends in order to identify and create tools to predict patients most at risk for readmission [1]. This is especially important in the future bundled payment system where readmission will be a measure of quality and where orthopedic surgeons may be penalized for readmissions. Moreover, by assessing patients' risk for readmission, physicians can both reduce the financial burden that surgical specialties place on hospitals and improve healthcare quality by creating appropriate healthcare plans for patients.

## Figures and Tables

**Table 1:** Demographic information for patients with tibia and femur fractures.

	Tibia (closed)	Tibia (open)	Tibia (total)	Femur (closed)	Femur (open)	Femur (total)
Average age (y)	38.1	34.3	36.1	33.83	31.98	33.37
Gender *n* (%)						
Male	56 (74.7)	71 (87.7)	127 (81.4)	267 (72.8)	97 (79.5)	367 (74.4)
Female	19 (25.3)	10 (12.3)	29 (18.6)	100 (27.2)	25 (20.5)	125 (25.6)
Average initial length of stay (d)	2.66	3.05	2.86	6.0	4.19	5.55
Gustilo grade *n* (%)						
1	N/A	15 (18.5)	N/A	N/A	38 (31.1)	N/A
2		37 (45.7)			43 (35.2)	
3		29 (35.8)			41 (33.6)	
4		0 (0.0)			0	
5		0 (0.0)			0	
ASA class *n* (%)						
1	18 (24.0)	15 (18.5)	33 (21.2)	32 (8.7)	9 (7.4)	41 (8.4)
2	48 (64.0)	57 (70.4)	105 (67.3)	218 (59.4)	71 (58.2)	289 (59.1)
3	8 (10.7)	8 (9.9)	16 (10.3)	96 (26.2)	31 (25.4)	127 (26.0)
4	1 (1.3)	1 (1.2)	2 (1.2)	21 (5.72)	11 (9.0)	32 (6.5)
5	0 (0.0)	0 (0.0)	0 (0.0)	0 (0)	0 (0.0)	0 (0.0)
Race *n* (%)						
African American	11 (14.7)	19 (23.5)	30 (19.2)	70 (19.1)	19 (15.6)	89 (18.2)
American Indian	0 (0.0)	0 (0.0)	0 (0.0)	0 (0.0)	1 (0.8)	1 (0.2)
Asian	0 (0.0)	0 (0.0)	0 (0.0)	3 (0.8)	1 (0.8)	4 (0.8)
Caucasian	59 (78.7)	56 (69.1)	115 (73.7)	263 (71.7)	92 (75.4)	355 (72.6)
Hispanic	3 (4.0)	4 (4.9)	7 (4.5)	12 (3.3)	5 (4.1)	17 (3.5)
Unknown	2 (2.7)	2 (2.5)	4 (2.6)	19 (5.2)	4 (3.3)	23 (4.7)

**(a):** 

Tibia	*n* (%)	30-Day readmission (0–30 days)				60-Day∗ readmission (0–60 days)				90-Day readmission (0–90 days)			
		Total	Infection	Surgical	Medical	Total	Infection	Surgical	Medical	Total	Infection	Surgical	Medical
Closed	75	4	2	2	0	**6 **	**3 **	**3**	**0**	6	3	3	0
	48.1%	5.3%	50.0%	50.0%	0.0%	**8.0%**	**50.0%**	**50.0%**	**0.0%**	8.0%	50.0%	50.0%	0.0%

Open	81	6	6	0	0	**12**	**11**	**1**	**0**	14	12	2	0
	51.9%	7.4%	100.0%	0.0%	0.0%	**14.8%**	**91.7%**	**8.3%**	**0.0%**	17.3%	85.7%	14.3%	0.0%

*p*		*p* = 0.6				***p* = 0.037** ^*^			*p* = 0.084				

**(b):** 

Tibia	*n* (%)	30-Day readmission (0–30 days)				60-Day readmission (0–60 days)				90-Day readmission (0–90 days)			
		Total	Infection	Surgical	Medical	Total	Infection	Surgical	Medical	Total	Infection	Surgical	Medical
Closed	367	7	0	2	5	23	0	3	20	27	1	3	23
	75.1%	1.9%	0.0%	28.6%	71.4%	6.3%	0.0%	13.0%	87.0%	7.4%	3.7%	11.1%	85.2%

Open	122	5	1	1	3	12	2	3	7	13	3	3	7
	24.9%	4.1%	20.0%	20.0%	60.0%	9.8%	16.7%	25.0%	58.3%	10.7%	23.1%	23.1%	53.8%

*p*		*p* = 0.175				*p* = 0.185			*p* = 0.249				

“Infection” refers to postoperative infection, “surgical” refers to postoperative surgical revision, and “medical” refers to nonoperative medical conditions such as UTIs, PNAs, hypotension, and anemia. ^*^There is a significant difference between the 60-day readmission rate for patients with closed tibia fractures and patients with open tibia fractures.

## Disclaimer

This study used no previously copyrighted materials or signed patient consent forms.

## References

[1] Medicare Advisory Payment Commission (2013). *Report to the Congress: Medicare and the Health Care Delivery System*.

[2] Lamberts H., Hofmans-Okkes I. (1996). Episode of care: a core concept in family practice. *Journal of Family Practice*.

[3] Jacobs J., Anderson G., Bell J. (2008). *The Burden of Musculoskeletal Diseases in the United States*.

[4] Burge R., Dawson-Hughes B., Solomon D. H., Wong J. B., King A., Tosteson A. (2007). Incidence and economic burden of osteoporosis-related fractures in the United States, 2005–2025. *Journal of Bone and Mineral Research*.

[5] Burge R. T., King A. B., Balda E., Worley D. (2003). Methodology for estimating current and future burden of osteoporosis in state populations: application to Florida in 2000 through 2025. *Value in Health*.

[6] Nacke E., Ramos N., Stein S., Hutzler L., Bosco J. A. (2013). When do readmissions for infection occur after spine and total joint procedures?. *Clinical Orthopaedics and Related Research*.

[7] Hahnel J., Burdekin H., Anand S. (2009). Re-admissions following hip fracture surgery. *Annals of the Royal College of Surgeons of England*.

[8] Costantino M. E., Frey B., Hall B., Painter P. (2013). The influence of a postdischarge intervention on reducing hospital readmissions in a medicare population. *Population Health Management*.

[9] Jordan C. J., Goldstein R. Y., Michels R. F., Hutzler L., Slover J. D., Bosco J. A. (2012). Comprehensive program reduces hospital readmission rates after total joint arthroplasty. *The American Journal of Orthopedics*.

[10] Brattström O., Eriksson M., Larsson E., Oldner A. (2014). Socio-economic status and co-morbidity as risk factors for trauma. *European Journal of Epidemiology*.

[11] Zhou X., Olivier J., McDaniel D. O. (2009). Impact of socioeconomic disparities and education on trauma-induced clinical complications. *Public Administration and Management*.

[12] Rüedi T. P., Buckley R. E., Moran C. G. (2007). *AO Principles of Fracture Management*.

